# The role of exopolysaccharides Psl and Pel in resistance of *Pseudomonas aeruginosa* to the oxidative stressors sodium hypochlorite and hydrogen peroxide

**DOI:** 10.1128/spectrum.00922-24

**Published:** 2024-08-28

**Authors:** Waleska S. da Cruz Nizer, Kira N. Allison, Madison E. Adams, Mario A. Vargas, Duale Ahmed, Carole Beaulieu, Deepa Raju, Edana Cassol, P. Lynne Howell, Joerg Overhage

**Affiliations:** 1 Department of Health Sciences, Carleton University, Ottawa, Ontario, Canada; 2 Program in Medicine, Research Institute, The Hospital for Sick Children, Toronto, Ontario, Canada; 3 Department of Biochemistry, University of Toronto, Toronto, Ontario, Canada; The Pennsylvania State University, University Park, Pennsylvania, USA

**Keywords:** *Pseudomonas aeruginosa*, biofilms, exopolysaccharides, oxidative stress, sodium hypochlorite, hydrogen peroxide, reactive chlorine species

## Abstract

*Pseudomonas aeruginosa* is well-known for its antimicrobial resistance and the ability to survive in harsh environmental conditions due to an abundance of resistance mechanisms, including the formation of biofilms and the production of exopolysaccharides. Exopolysaccharides are among the major components of the extracellular matrix in biofilms and aggregates of *P. aeruginosa*. Although their contribution to antibiotic resistance has been previously shown, their roles in resistance to oxidative stressors remain largely elusive. Here, we studied the function of the exopolysaccharides Psl and Pel in the resistance of *P. aeruginosa* to the commonly used disinfectants and strong oxidizing agents NaOCl and H_2_O_2_. We observed that the simultaneous inactivation of Psl and Pel in *P. aeruginosa* PAO1 mutant strain ∆*pslA pelF* resulted in a significant increase in susceptibility to both NaOCl and H_2_O_2_. Further analyses revealed that Pel is more important for oxidative stress resistance in *P. aeruginosa* and that the form of Pel (i.e., cell-associated or cell-free) did not affect NaOCl susceptibility. Additionally, we show that Psl/Pel-negative strains are protected against oxidative stress in co-culture biofilms with *P. aeruginosa* PAO1 WT. Taken together, our results demonstrate that the EPS matrix and, more specifically, Pel exhibit protective functions against oxidative stressors such as NaOCl and H_2_O_2_ in *P. aeruginosa*.

**IMPORTANCE:**

Biofilms are microbial communities of cells embedded in a self-produced polymeric matrix composed of polysaccharides, proteins, lipids, and extracellular DNA. Biofilm bacteria have been shown to possess unique characteristics, including increased stress resistance and higher antimicrobial tolerance, leading to failures in bacterial eradication during chronic infections or in technical settings, including drinking and wastewater industries. Previous studies have shown that in addition to conferring structure and stability to biofilms, the polysaccharides Psl and Pel are also involved in antibiotic resistance. This work provides evidence that these biofilm matrix components also contribute to the resistance of *Pseudomonas aeruginosa* to oxidative stressors including the widely used disinfectant NaOCl. Understanding the mechanisms by which bacteria escape antimicrobial agents, including strong oxidants, is urgently needed in the fight against antimicrobial resistance and will help in developing new strategies to eliminate resistant strains in any environmental, industrial, and clinical setting.

## INTRODUCTION

*Pseudomonas aeruginosa* is a Gram-negative, opportunistic pathogen found in various environments, from water and soil to animals and humans. This bacterium is commonly associated with nosocomial and difficult-to-treat infections, mainly in immune-compromised individuals (1, 2). The high versatility of *P. aeruginosa*, its increased resistance to several classes of antibiotics, and its ability to form robust biofilms led this bacterium to be considered a critical priority pathogen that poses a great threat to human health (1, 3).

Biofilms are the most common form of bacterial growth in nature. They are cellular aggregates that can either be found in suspension or attached to biotic or abiotic surfaces encased by a self-produced extracellular polymeric matrix. This extracellular polymeric substance (EPS) matrix, which accounts for over 90% of the biofilm biomass (4), is primarily comprised of polysaccharides, extracellular DNA (eDNA), proteins, and lipids and provides the infrastructure for an extremely versatile and adaptable form of multicellular microbial life (5). Besides surface-attached biofilms, non-attached aggregates have been described in nature and disease (6), with research showing that the physiological characteristics of bacteria within such aggregates closely resemble those found in surface-associated biofilms, leading to the classification of aggregates as biofilms (7). Biofilms protect bacteria against the host immune system and render them more tolerant to antibiotics and disinfectants than their planktonic counterparts. This reduced susceptibility is attributed to several factors, including the EPS, high cell density, heterogeneity in metabolism, low metabolic activity, and the presence of persister cells (8, 9).

Exopolysaccharides are highly diverse in composition and structure and are thought to be the major and most important component of the EPS. Different bacterial species produce different exopolysaccharides, with some microbes producing multiple polysaccharides at different stages of aggregate and biofilm formation (5). *P. aeruginosa* produces at least three exopolysaccharides: Psl, Pel, and alginate (10). Psl, synthesized by the *pslA-O* operon, is a neutrally charged mannose-rich polysaccharide involved in initial cell-to-cell and cell-surface attachment and is important for the initial attachment of the cells as well as maintenance of the mature biofilm structure (11–14). Pel, expressed through the *pelA-G* operon, is a positively charged polysaccharide composed of α−1,4 linked galactosamine and *N-*acetylgalactosamine repeats and has been shown to play roles in cell-to-cell interactions, acting as a primary structural scaffold for the microbial community (15, 16). Psl and Pel are essential for microcolony formation during the early stages of biofilm development (17). In contrast to Psl and Pel, alginate is a negatively charged polymer not required for initial attachment. However, it is important for later stages of biofilm development and a mucoid phenotype (18, 19). Alginate is only produced by some strains of *P. aeruginosa* and is not a critical matrix component of the laboratory strain *P. aeruginosa* PAO1 (20, 21).

Besides their roles as structural elements, exopolysaccharides have been linked to increased tolerance against antimicrobial agents, including antibiotics and disinfectants. Psl has been shown to promote resistance of *P. aeruginosa* PAO1 biofilms to several classes of antibiotics, particularly at the early stages of biofilm formation (21, 22), whereas Pel enhances *P. aeruginosa* resistance against aminoglycoside antibiotics (15).

Oxidative antimicrobial agents [e.g., hydrogen peroxide (H_2_O_2_) and sodium hypochlorite (NaOCl)] have been commonly used to eradicate microbes. Among them, NaOCl and its active ingredient, hypochlorous acid (HOCl), are potent and fast-acting reactive chlorine species (RCS) widely used in sanitation and disinfection purposes in industrial, domestic, and hospital settings. Furthermore, HOCl is also produced by the human immune system as a defense against invading pathogens (23, 24). The broad-spectrum antimicrobial action of NaOCl is due to its interaction with proteins, nucleic acids, and lipids, affecting diverse cellular processes, such as membrane stabilization, protein and DNA synthesis, ATP production, and protein transport through the membrane (24).

In the present study, we analyzed the roles of exopolysaccharides in the tolerance of *P. aeruginosa* PAO1 to the oxidative stressors NaOCl and H_2_O_2_. Since alginate is not a critical EPS component in *P. aeruginosa* PAO1 (21, 25), we focused our analyses on the polysaccharides Psl and Pel. Using a set of PAO1 *∆pslA*, *∆pelF,* and *∆pslA pelF* mutants, we show that the absence of both polysaccharides leads to a 2.5-fold and 8-fold increase in susceptibility toward NaOCl and H_2_O_2_, respectively. Furthermore, we found that Pel plays a more significant role in NaOCl resistance than Psl.

## RESULTS

### Absence of Psl and Pel increases susceptibility of *P. aeruginosa* PAO1 to NaOCl

**Fig 1:**
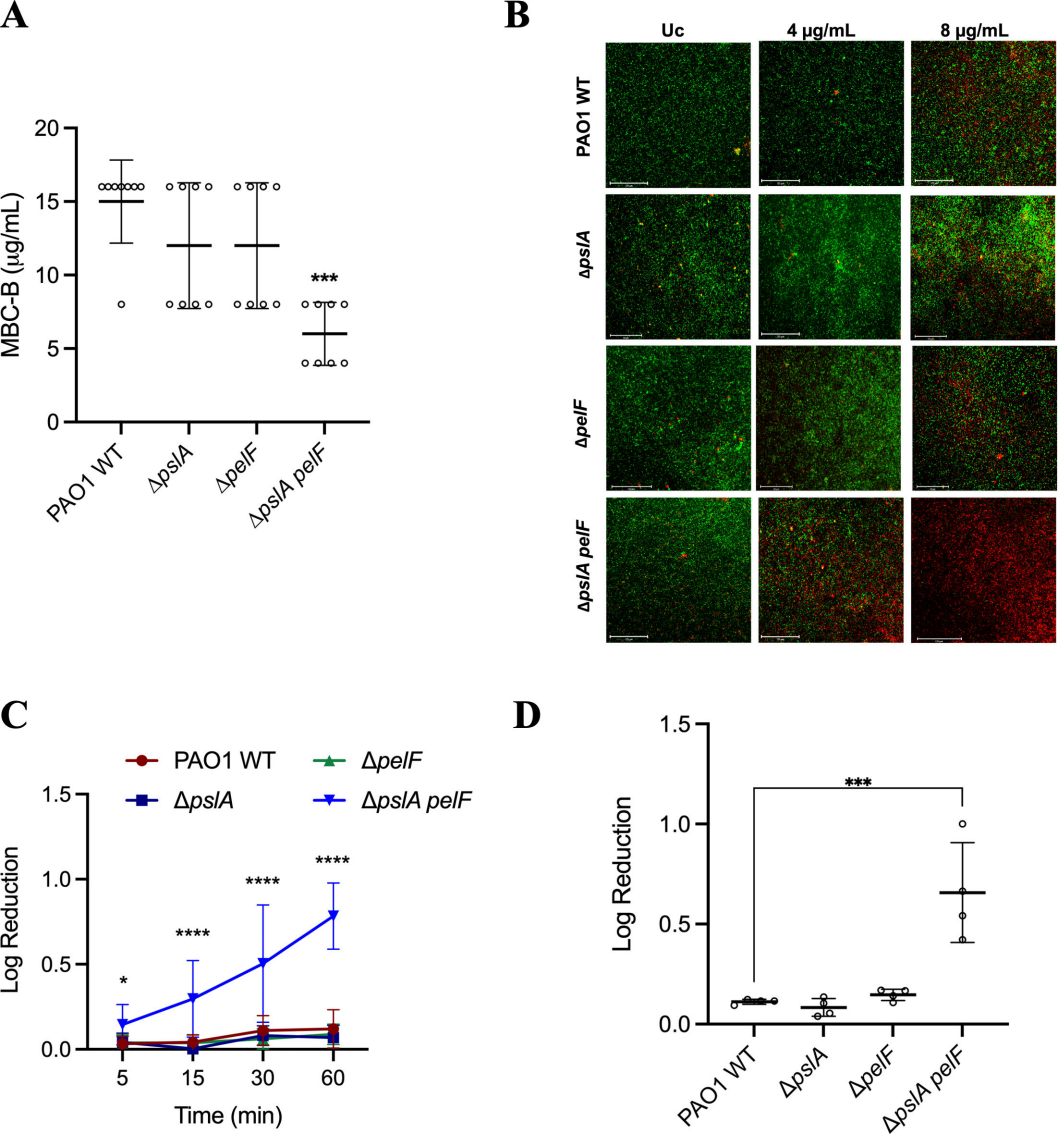
Susceptibility of PAO1 WT and PAO1 ∆*pslA*, ∆*pelF*, and ∆*pslA pelF* to NaOCl. PAO1 WT and mutant strains were grown in polystyrene 96- or 12-well microplates for 24 h at 37°C in BM2 biofilm medium under static conditions and treated with NaOCl. (**A**) Susceptibility was evaluated by the MBC-B assay. (**B**) Fluorescence microscopy of PAO1 WT and ∆*pslA,* ∆*pelF,* and ∆*pslA pelF* mutant biofilms treated with 4 µg/mL and 8 µg/mL NaOCl. Biofilms were grown in flat-bottom polystyrene 96-well microplates, treated with NaOCl at 4 and 8 µg/mL NaOCl 1 h at 37°C under static conditions, and stained with DNA-intercalating 1:1 Syto9 and PI dyes and visualized by fluorescence microscopy at 20× magnification. Untreated biofilms were used as controls. Pictures are representative of three independent experiments with three replicates. Scale bars represent 125 µm. (**C**) Time kill kinetics of PAO1 WT, ∆*pslA*, ∆*pelF*, and ∆*pslA pelF* biofilms treated with 8 µg/mL NaOCl for 5, 15, 30, and 60 min. (**D**) Susceptibility of PAO1 WT and ∆*pslA*, ∆*pelF*, and ∆*pslA pelF* mutant strains of biofilms using the agar colony biofilm model. *P. aeruginosa* PAO1 WT, ∆*pslA,* ∆*pelF,* and ∆*pslA pelF* were grown overnight in LB media, harvested by centrifugation, washed twice with PBS, and the OD_600nm_ was adjusted to 0.05 (5 × 10^7^ CFU/mL). Ten microliters of bacterial culture were inoculated in the center of the semi-permeable membranes, and the plates were incubated for 48 h at 37°C to allow biofilm establishment. Colony biofilms were treated with NaOCl at 32 µg/mL, which were incubated for 1 h at 37°C and plated following the drop plate method. Data represent the mean ± standard deviation of at least three independent experiments. Data was analyzed by one-way ANOVA. *P < 0.05; ***P < 0.001; ****P < 0.0001. Uc: untreated control.

In order to study the involvement of Psl and Pel in NaOCl resistance, we performed the minimal bactericidal concentration in biofilms (MBC-B) assay in microtiter plates, as previously described (26), and challenged 24 h grown, static cultures of PAO1 wild-type (WT) and mutant strains ∆*pslA,* ∆*pelF,* and ∆*pslA pelF* with increasing concentrations of NaOCl in the range of 2–1024 µg/mL (Fig. 1A). Of note, although the WT and ∆*pelF* mutant strains form similar biofilms under these growth conditions, ∆*pslA* and ∆*pslA pelF* show biofilm defects in flow chamber experiments and form only monolayer biofilms with the absence of microcolonies and reduced biomass compared with WT (25). This indicates that the cells analyzed in this particular assay likely represent a mixture of biofilm cells as well as loosely attached and sedimented cells. After removing planktonic cells and subsequent NaOCl treatment, we observed that 15 µg/mL of NaOCl was required to kill PAO1 WT cells. In contrast, only 6 µg/mL NaOCl was needed to eliminate cells of ∆*pslA pelF*, indicating a 2.5-fold increase in susceptibility. Both single-mutant ∆*pslA* and ∆*pelF* exhibited an MBC-B of 12 µg/mL, which was not statistically significant compared with the MBC-B of PAO1 WT.

Since the ∆*pslA* and ∆*pslA pelF* mutants are known to have reduced biofilm biomass compared with the WT (15), we determined whether the increased susceptibility of ∆*pslA pelF* was due to the overall lower biomass of this mutant strain compared with the WT rather than to the effect of NaOCl. In accordance with (21, 21), we conducted an MBC-B experiment for cells grown for 6 h (attachment, early-stage biofilms, and lower biofilm biomass) and 24 h (mature biofilms and higher biofilm biomass) by treating the cells with NaOCl (2–1,024 µg/mL) for 1 h. For all tested strains, we received no statistically significant changes in the MBC-B for cells grown for 6 h and 24 h (Fig. S1). These results indicate that the simultaneous loss of Psl and Pel increases the susceptibility of *P. aeruginosa* to NaOCl, and this increase in susceptibility is not due to lower biomass.

In contrast, analyses of planktonic cells revealed no differences in susceptibility and growth of PAO1 WT, ∆*pslA,* ∆*pelF,* and ∆*pslA pelF* in the presence of 0.5, 1, and 2 µg/mL NaOCl (Table S1). Moreover, no differences in cell viability by CFU determination were obtained upon treatment of planktonic cells with 1.25 µg/mL NaOCl (Fig. S2).

### Fluorescence microscopy, kill-curve kinetics, and agar colony model confirm the ∆*pslA pelF* phenotype

To further verify these results, we assessed the viability of *P. aeruginosa* PAO1 WT, ∆*pslA,* ∆*pelF,* and ∆*pslA pelF* after a 1 h treatment with NaOCl using fluorescence microscopy in combination with LIVE/DEAD cell viability staining as well as time kill-curve kinetics. In these experiments, we added a washing step after initial growth and before treatment to remove all non-surface-adhered cells. As shown in Fig. 1B, treatment of PAO1 WT, ∆*pslA,* and ∆*pelF* with a sub-MBC-B concentration of NaOCl of 4 µg/mL did not affect cell viability, and the number of dead cells (red) in all three strains was low and comparable with the untreated controls. Furthermore, treatment with 8 µg/mL NaOCl also revealed a similar phenotype for PAO1 WT, ∆*pslA,* and ∆*pelF* with a decrease in cell viability; however, many cells were still not affected. In contrast, most ∆*pslA pelF* double mutant cells were inactivated at 4 µg/mL NaOCl, and almost all cells were killed after treatment with 8 µg/mL (Fig. 1B). The reduction in the amount of live cells in the NaOCl-treated conditions, expressed in relative fluorescence units (RFU), was normalized based on the untreated control and is shown in Fig. S3a.

We then analyzed time kill-curve kinetics of adhered cells by determination of CFU numbers after NaOCl treatment over time (Fig. 1C) to show that ∆*pslA pelF* is more susceptible to NaOCl treatment in comparison to the WT and single knockout mutants. NaOCl at 8 µg/mL induced a significant reduction in the viable cell counts of the ∆*pslA pelF* double mutant (i.e., 0.15-fold, 0.3-fold, 0.5-fold, and 0.78-fold reduction in the log CFU after 5, 15, 30, and 60 min, respectively), being the most pronounced effect. PAO1 WT, ∆*pslA,* and ∆*pelF* did not present increased susceptibility to NaOCl treatment. After 60 min, a log reduction of 0.12, 0.07, and 0.09 was obtained for PAO1 WT, ∆*pslA* and ∆*pelF*, respectively.

Biofilms can be studied through many different methods. Therefore, to further confirm the results previously obtained, we utilized an agar colony biofilm model to evaluate the susceptibility of PAO1 WT, ∆*pslA,* ∆*pelF,* and ∆*pslA pelF* to NaOCl. This model, in which bacteria are grown as biofilms on the surface of an agar plate, has been commonly used to evaluate the effect of antimicrobial agents (27, 28). PAO1 WT, ∆*pslA,* ∆*pelF,* and ∆*pslA pelF* were grown on a polycarbonate membrane filter for 48 h at 37°C before being treated with NaOCl at 32 µg/mL for 1 h. This concentration represents a sub-lethal concentration of NaOCl found for the PAO1 WT strain in this biofilm model. In accordance with our initial microtiter plate experiments, the ∆*pslA pelF* double mutant presented an increased susceptibility to NaOCl compared with PAO1 WT, ∆*pslA,* and ∆*pelF* strains (Fig. 1D). A log reduction of 0.66 in the number of viable cells was obtained for the ∆*pslA pelF* after treatment with NaOCl, whereas PAO1 WT, ∆*pslA,* and ∆*pelF* presented a non-statistical reduction in cell viability of 0.11, 0.08, and 0.14, respectively (Fig. 1D).

Together, these results show that the simultaneous lack of Psl and Pel significantly increases the susceptibility of PAO1 to NaOCl.

### Psl and Pel play additional roles in H_2_O_2_ susceptibility

**Fig 2:**
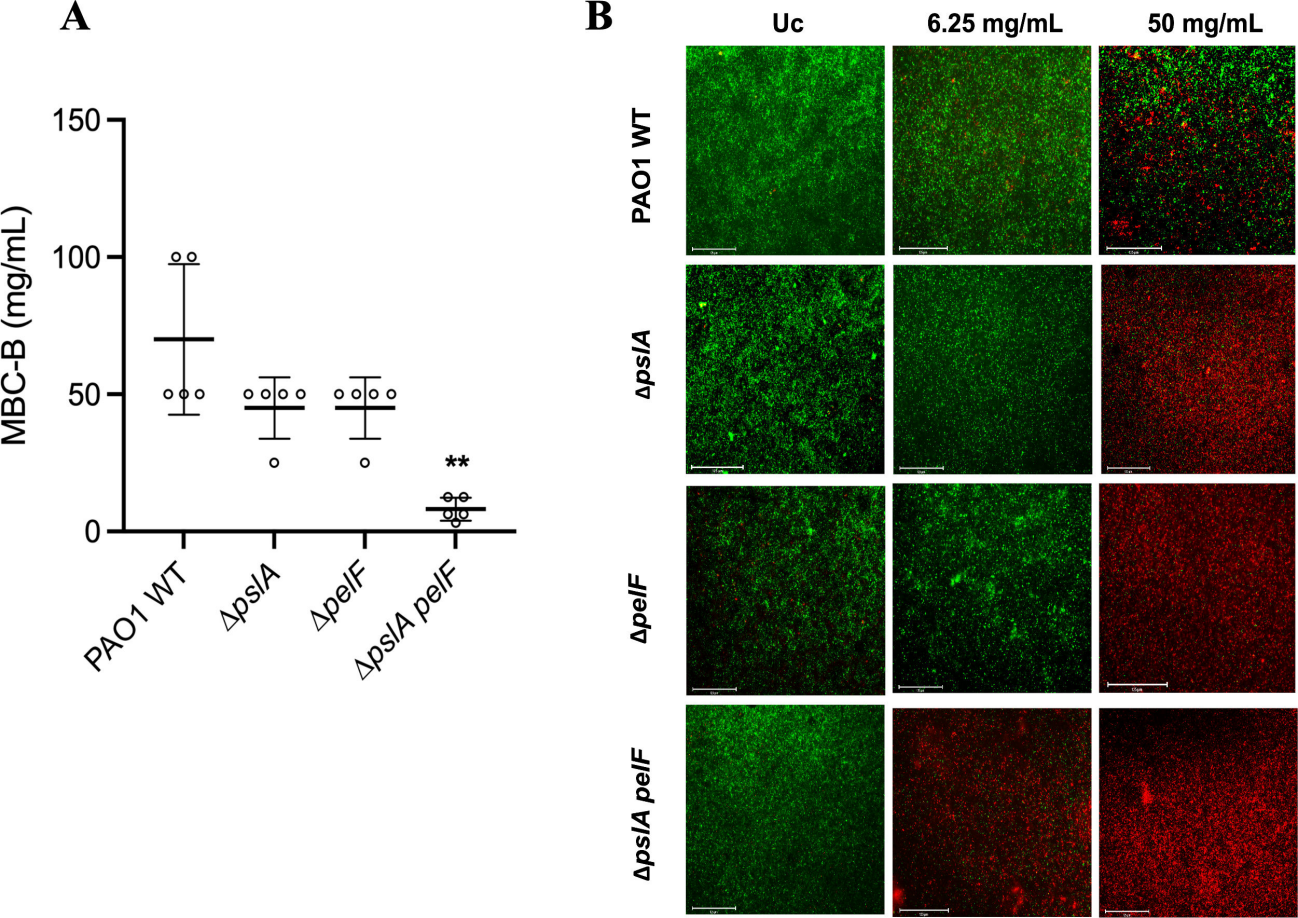
Susceptibility of PAO1 WT and PAO1 ∆*pslA*, ∆*pelF*, and ∆*pslA pelF* biofilms to H_2_O_2_. PAO1 WT and mutant strains were grown in polystyrene 96- or 12-well microplates for 24 h at 37°C in BM2 biofilm medium under static conditions and treated with H_2_O_2_. (**A**) Susceptibility was evaluated by the MBC-B assay. (**B**) Microscopy pictures of PAO1 WT and ∆*pslA,* ∆*pelF,* and ∆*pslA pelF* mutant biofilms treated with 6,250 µg/mL and 50,000 µg/ml H_2_O_2_. Biofilms were grown for 24 h at 37°C in BM2 biofilm medium in flat-bottom polystyrene 96-well microplates. H_2_O_2_ was diluted in BM2, and biofilms were treated for 1 h at 37°C under static conditions. Biofilms were stained with DNA-intercalating 1:1 Syto9 and PI dyes and visualized by fluorescence microscopy at 20× magnification. Untreated biofilms were used as the positive control. Pictures are representative of three independent experiments with three replicates each. Scale bars represent 125 µm. Data represent the mean ± standard deviation of at least three independent experiments. Data was analyzed by one-way ANOVA. **P < 0.01. Uc: untreated control.

To evaluate whether the susceptibility phenotype obtained is hypochlorite-specific or if Psl and Pel are involved in a more general response to oxidative stressors, we performed MBC-B analyses in the presence of varying concentrations of H_2_O_2_ (1.95–100 mg/mL) to show that cells of ∆*pslA* ∆*pelF* were also more susceptible to this oxidant. As shown in Fig. 2A, PAO1 WT exhibited an MBC-B of 70 mg/mL, which was more than 8-fold more resistant to H_2_O_2_ treatment compared with ∆*pslA pelF* with an MBC-B of 8.125 mg/mL. A less pronounced and not statistically significant increase in susceptibility to H_2_O_2_ was detected for ∆*pslA* and ∆*pelF*, with an MBC-B of 45 mg/mL for both strains.

In addition, we used fluorescence microscopy and LIVE/DEAD staining to analyze cell viability after the treatment with H_2_O_2_ at 6.25 and 50 mg/mL H_2_O_2_ for 1 h. Treatment with 6.25 mg/mL H_2_O_2_ killed nearly all cells of ∆*pslA pelF*. However, PAO1 WT, ∆*pslA,* and ∆*pelF* cells were not affected by this concentration of H_2_O_2_. Treatment with 50 mg/mL H_2_O_2_ revealed the complete killing of ∆*pslA*, ∆*pelF,* and ∆*pslA pelF*, whereas PAO1 WT still showed a significant number of living cells (Fig. 2B). The reduction in the number of live cells (green) in the NaOCl-treated conditions, expressed in relative fluorescence units (RFU), was normalized based on the untreated control and is shown in Fig. S3b. These data are in accordance with our results obtained for NaOCl, suggesting that Psl and Pel provide protection against different oxidative stressors.

### Psl and Pel protects ∆*pslA pelF* and *Enterococcus faecalis* against oxidizing agents

In their natural environment, biofilms are predominantly found as heterogeneous and often multi-species populations, which confer many advantages to the bacteria, including the ability to share resources and increased resistance to antimicrobial treatment (29). With this in mind, we evaluated if Psl and Pel produced by the PAO1 WT could influence the survival of the Psl- and Pel-deficient strain ∆*pslA pelF* as well as *E. faecalis*, an opportunistic Gram-positive pathogen often isolated together with *P. aeruginosa* from biofilm infections (30).

**Fig 3:**
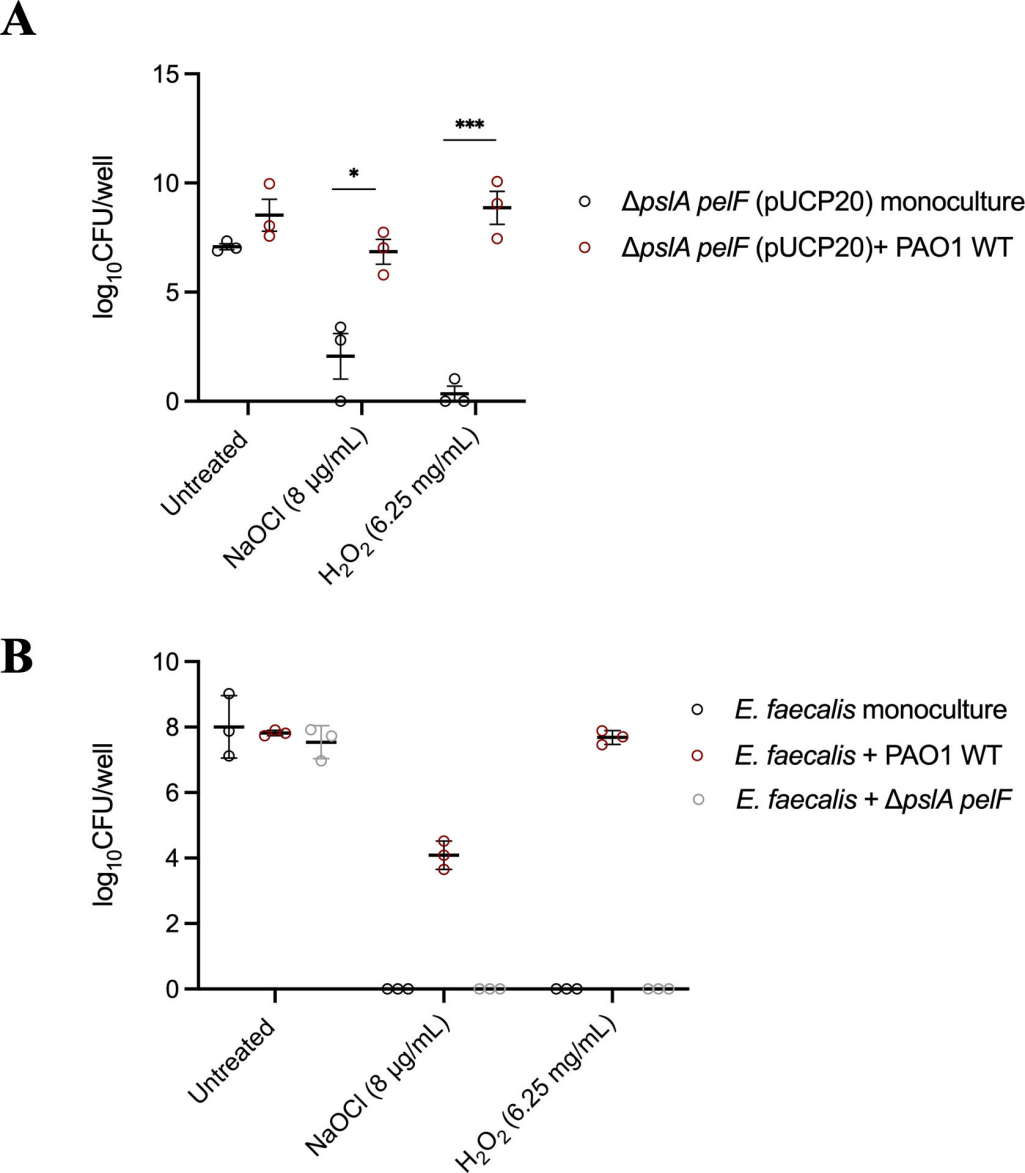
Viability of (**A**) ∆*pslA pelF* and (**B**) *E. faecalis* cells after the treatment with NaOCl and H_2_O_2_ as monoculture or mixed culture. (**A**) CFU numbers of ∆*pslA pelF* (pUCP20) cells after NaOCl and H_2_O_2_ treatment of mono- and co-culture biofilms with PAO1 WT (pJN105). Mono- and co-culture biofilms were grown for 24 h at 37°C in BM2 biofilm medium in flat-bottom polystyrene 12-well microplates. Biofilms were washed and treated with NaOCl or H_2_O_2_ diluted in BM2 at 8 µg/mL and 6.25 mg/mL, respectively, and biofilms were treated for 1 h at 37°C under static conditions. Then, sodium thiosulfate at 10 mM was added to NaOCl-treated biofilms, and CFU was determined by the drop method. For PAO1 WT (pJN105) was selected in LB agar plates supplemented with 30 µg/mL gentamicin, and ∆*pslA pelF* (pUCP20) cells were selected in LB agar plates supplemented with 300 µg/mL carbenicillin. (**B**) CFU numbers of *E. faecalis* cells after the treatment with NaOCl and H_2_O_2_ as monoculture or mixed culture with PAO1 WT or ∆*pslA pelF*. Mono- and co-culture biofilms were grown for 24 h at 37°C in DMEM in flat-bottom polystyrene 12-well microplates. Biofilms were washed and treated with NaOCl or H_2_O_2_ diluted in BM2 at 8 µg/mL and 6.25 mg/mL, respectively, and biofilms were treated for 1 h at 37°C under static conditions. Then, sodium thiosulfate at 10 mM was added to NaOCl-treated biofilms, and CFU was determined by the drop method. *P. aeruginosa* was selected using BM2 agar plates, and *E. faecalis* was selected using LB plates supplemented with 5 µg/mL gentamicin and 12 µg/mL Polymyxin B. Data represent the mean ± standard deviation of at least three independent experiments. Data was analyzed by one-way ANOVA or *t*-test for comparison between two groups. *P < 0.05; ***P < 0.001.

First, plasmid pJN105 (gentamicin resistance) was introduced into PAO1 WT and plasmid pUCP20 (carbenicillin resistance) into ∆*pslA pelF* in order to distinguish between both strains in our co-culture analyses. Biofilms formed by PAO1 WT (pJN105) and ∆*pslA pelF* (pUCP20) were then cultured in 12-well microplates in BM2 biofilm medium for 24 h at 37°C, treated with NaOCl at 8 µg/mL or H_2_O_2_ at 6.25 mg/mL for 1 h, and susceptibility was determined by CFU counts. These analyses showed an increase in the amount of viable ∆*pslA pelF* (pUCP20) cells when this strain was co-cultured together with PAO1 WT (pJN105) (Fig. 3A). For NaOCl treatment, a 5-fold increase in the number of viable cells was obtained, whereas for H_2_O_2_, the co-culture of PAO1 WT (pJN105) with ∆*pslA pelF* (pUCP20) increased the viability of the mutant by 25-fold.

The co-culture of *P. aeruginosa* with *E. faecalis* was performed using a co-culture model developed in our lab, in which biofilms were grown for 24 h at 37°C in DMEM, *P. aeruginosa* was selected using BM2 agar plates, and *E. faecalis* was selected using LB agar plates supplemented with 5 µg/mL gentamicin and 12 µg/mL polymyxin B. When grown as a mono-species biofilm, no *E. faecalis* cells were detected after the treatment with NaOCl and H_2_O_2_, whereas in the co-culture together with PAO1 WT, *E. faecalis* was able to survive the toxic effect of both oxidizing agents, presenting log_10_ CFU/well of 4 and 7, respectively (Fig. 3B). On the other hand, the mixed culture biofilm formed by ∆*pslA pelF* and *E. faecalis* did not affect the survival rate of *E. faecalis* to NaOCl and H_2_O_2_ stress, in which, as in the monoculture, no viable cells were detected after the 1 h treatment with these oxidants.

These results indicate that the exopolysaccharides Psl and Pel produced by *P. aeruginosa* protect Psl/Pel-negative strains of *P. aeruginosa* as well as *E. faecalis* against NaOCl and H_2_O_2_ stress.

### Pel increases the resistance of *P. aeruginosa* PAO1 to NaOCl

**Fig 4:**
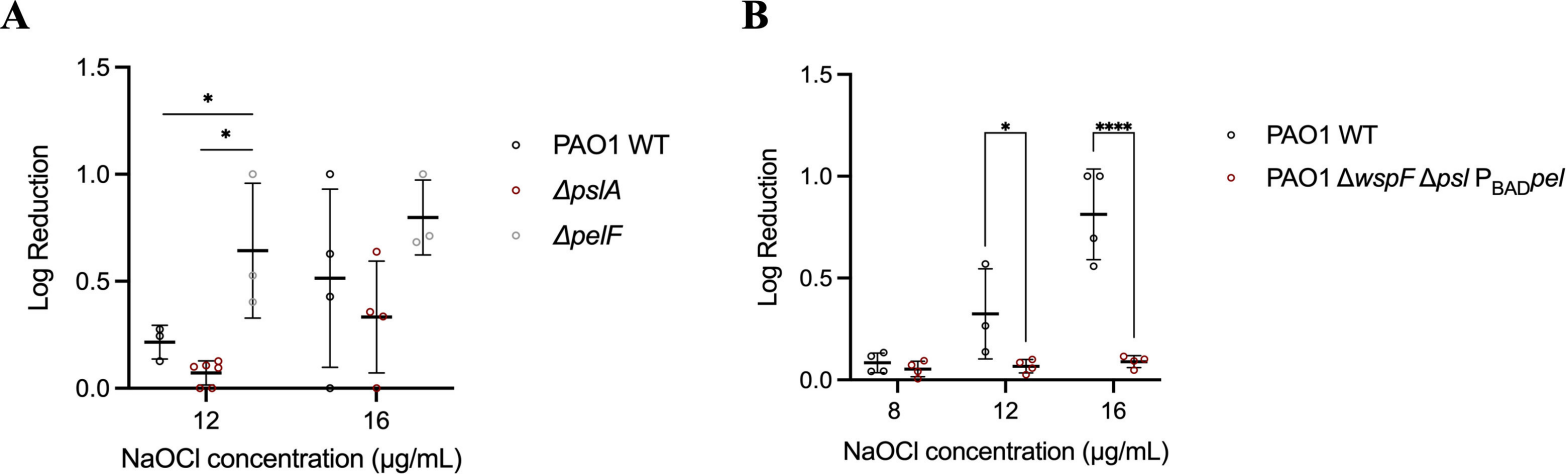
Susceptibility of (**A**) PAO1 WT and PAO1 mutants ∆*pslA* and ∆*pelF* and (**B**) PAO1 Δ*wspF* Δ*psl* P_BAD_*pel* biofilms to NaOCl. PAO1 WT and mutant strains were grown in polystyrene 12-well microplates for 24 h at 37°C in BM2 biofilm medium under static conditions and treated with NaOCl at 8, 12, or 16 µg/mL. Then, 10 mM sodium thiosulfate was added to quench the toxic effect of the remaining NaOCl, and CFU was determined by the drop method. Data represent the mean ± standard deviation of at least three independent experiments. Data were analyzed by one-way ANOVA or *t*-test for comparison between two groups. *P < 0.05; ****P < 0.0001.

To further investigate potential differences between Psl and Pel in resistance of *P. aeruginosa* to NaOCl, we analyzed cell viability of the PAO1 ∆*pslA* and ∆*pelF* mutant strains treated with NaOCl at 12 and 16 µg/mL, respectively, for 1 h. A log reduction of 0.64 in cell viability was observed for the treatment of *P. aeruginosa* ∆*pelF* with 12 µg/mL NaOCl, whereas for PAO1 WT and ∆*pslA,* a reduction of 0.21 and 0.071 in the log of CFU/well, respectively, was obtained after the treatment with 12 µg/mL NaOCl for 1 h (Fig. 4A). No statistical difference was found for the treatment with 16 µg/mL.

Based on these results, we then analyzed if the overproduction of Pel would result in reduced susceptibility of *P. aeruginosa* to NaOCl. For this, the Pel overproducing strain PAO1 Δ*wspF* Δ*psl* P_BAD_*pel* (31) was treated with NaOCl at 8, 12, and 16 µg/mL for 1 h, and cell viability was assessed by CFU determination to show that Δ*wspF* Δ*psl* P_BAD_*pel* indeed exhibited increased tolerance to NaOCl in comparison to PAO1 WT (Fig. 4B).

### Pel increases the resistance of *P. aeruginosa* PA14 to NaOCl independently of its structure

**Fig 5:**
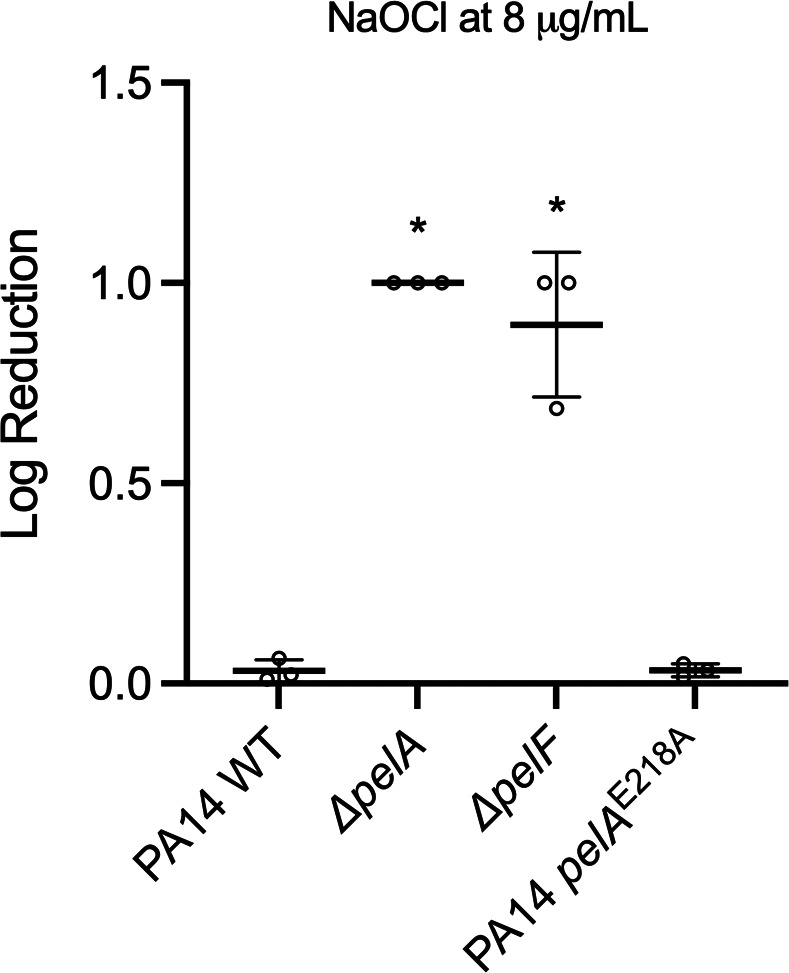
Susceptibility of PA14 WT and PA14 mutants ∆*pelA,* ∆*pelF,* and PA14 *pelA*^E218A^ biofilms to NaOCl. PAO1 WT and mutant strains were grown in polystyrene 96- or 12-well microplates for 24 h at 25°C in BM2 biofilm medium under static conditions and treated with NaOCl at 8 µg/mL. Then, 10 mM sodium thiosulfate was added to quench the toxic effect of the remaining NaOCl, and CFU was determined by the drop method. Data represent the mean ± standard deviation of at least three independent experiments. Data were analyzed by one-way ANOVA. *P < 0.05.

*P. aeruginosa* strain PA14 synthesizes Pel but does not produce Psl (32). With this in mind, we investigated if additional knockout of Pel in PA14 mutant strains ∆*pelA* and ∆*pelF* would attenuate NaOCl susceptibility in *P. aeruginosa* PA14. After treatment with 8 µg/mL NaOCl, we observed that PA14 ∆*pelA* and ∆*pelF* presented a 1 and 0.89 log reduction in cell viability, respectively, which was 33-fold more killing in comparison to cells of PA14 WT (Fig. 5).

As Pel can be found in both cell-associated and cell-free forms (33), we investigated if the localization of the polymer played differential roles in NaOCl resistance. For this, we tested the PA14 *pelA*^E218A^ strain, a mutant in which the PelA hydrolase activity was disrupted by using the inactive catalytic point variant E218A (34). In the absence of hydrolase activity, Pel was found almost exclusively in the cell-associated fraction in the PAO1 Pel overexpressing strain compared with the parental strain (34). As shown in Fig. 5, cell viability analyzed by CFU determination upon treatment with 8 µg/mL NaOCl for 1 h resulted in a 0.033 reduction in the cell viability of PA14 *pelA*^E218A^, a phenotype similar to that obtained for the PA14 WT (0.032 log reduction). The PA14 *pelA*^E218A^ analysis suggests that the distribution of Pel between the two forms (i.e., cell-associated and cell-free) does not influence the NaOCl susceptibility of PA14.

### Treatment of PAO1 biofilms with glycoside hydrolases PslG_h_ and PelA_h_ does not affect NaOCl susceptibility

**Fig 6:**
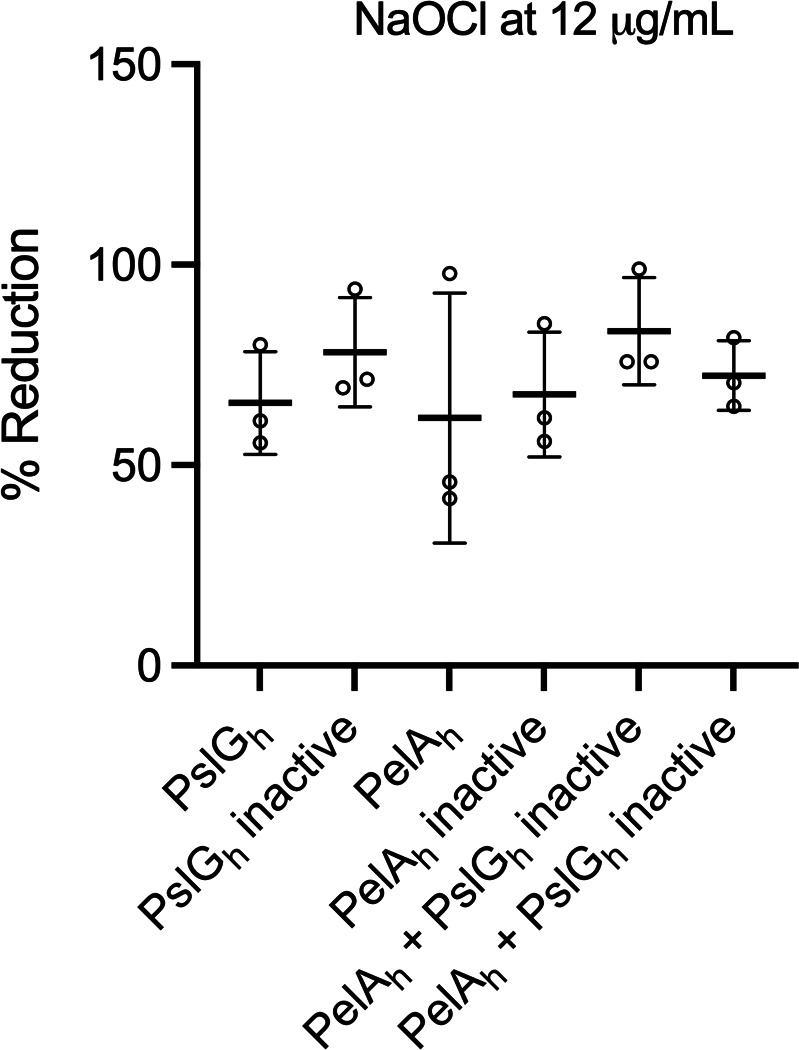
NaOCl susceptibility of PAO1 WT biofilms treated with the glycoside hydrolases PslG_h_ and PelA_h_. PAO1 WT biofilms were grown in polystyrene 12-well microplates for 24 h at 37°C in BM2 biofilm medium under static conditions and treated with 2 µM of PslG_h_, PelA_h_, or a combination of PslG_h_ and PelA_h_ for 1 h at 25°C. Then, NaOCl at 12 µg/mL was added for 1 h, followed by 10 mM sodium thiosulfate to quench the toxic effect of the remaining NaOCl, and CFU was determined by the drop method. Heat-inactive enzymes were used as a control. Data represent the mean ± standard deviation of at least three independent experiments. Data were analyzed by one-way ANOVA.

Previous studies have shown that the glycoside hydrolases PslG and PelA (PslG_h_ and PelA_h_, respectively) degrade Psl and Pel, respectively (35), and contribute to the killing effect of antibiotics such as tobramycin, Polymyxin B, colistin, and neomycin (36). We, therefore, aimed to evaluate if treating PAO1 WT biofilms with PslG_h_ and PelA_h_ would influence the susceptibility of *P. aeruginosa* to NaOCl. First, hydrolase activities were confirmed by analyzing the biofilm biomass of PAO1 WT biofilms treated with active or heat-inactive hydrolases (Fig. S4). Then, to evaluate the effect of the hydrolases on NaOCl susceptibility, PAO1 WT biofilms were treated with 2 µM of PslG_h_, PelA_h_, or the combination of PslG_h_ and PelA_h_ for 1 h, followed by treatment with NaOCl at 12 µg/mL for 1 h. Cell viability analyses revealed no statistical difference between the PslG_h_-, PelA_h_-, and PslG_h_ + PelA_h_-treated biofilms compared with those treated with inactive enzymes (Fig. 6). These results suggest that degraded polysaccharides also protect *P. aeruginosa* against NaOCl. In this context, upon degradation by hydrolases, polysaccharide fragments would still be available in the culture supernatant to react with NaOCl, as opposed to the results observed for the mutant strains, which lack the respective polysaccharides.

## DISCUSSION

Bacteria have developed a variety of mechanisms to resist the damage caused by oxidizing agents, such as the expression of detoxifying enzymes (e.g., catalase and peroxidase), protein and DNA repair systems, activation of transcriptional regulators (e.g., OxyR, SoxR, and OhrR), and the formation of biofilms (24, 37). Previously, we and others have shown that sub-lethal concentrations of oxidants such as NaOCl stimulate biofilm formation (38) and that this stimulation is mediated in several different ways, including changes in cell morphology (e.g., increasing membrane hydrophobicity) (39, 40) and synthesis of cyclic-di-GMP (c-di-GMP) and EPS matrix components, mainly exopolysaccharides (38, 41–43). In this study, we demonstrate that the simultaneous inactivation of the exopolysaccharides Psl and Pel in *P. aeruginosa* PAO1 mutant strain ∆*pslA pelF* resulted in increased susceptibility to oxidizing agents compared with the PAO1 WT and ∆*pslA* and ∆*pelF* single mutant strains (Fig. 1 and 2). The PAO1 mutant strains used in this study were previously characterized (44). Considering that cells of ∆*psl* and ∆*psl pel* mutants do not form mature biofilms but are still able to adhere to surfaces and form monolayer biofilms with reduced biomass (25), we used a variety of different methods and approaches to study the roles of Psl and Pel in oxidative stress resistance of *P. aeruginosa*. These experiments confirmed the increased susceptibility of the double mutant ∆*pslA pelF* to NaOCl stress compared with the WT and single-knockout mutant strains and showed that compared with Psl, Pel plays a more significant role in oxidative stress resistance in *P. aeruginosa* (Fig. 1).

Furthermore, since NaOCl resistance has been shown to be mediated by a combination of specific and general resistance mechanisms (24, 37, 45, 46), we also tested if the loss of Psl and Pel in *P. aeruginosa* PAO1 increases the susceptibility of biofilms to H_2_O_2_, a widely used reactive oxygen species (ROS) and a component of the innate immune system (47). As obtained for NaOCl, the absence of both polysaccharides in the ∆*pslA pelF* double mutant increased its susceptibility to H_2_O_2_ compared with the WT and single mutant strains (Fig. 2), indicating, therefore, that the exopolysaccharides of the biofilm matrix of *P. aeruginosa* function as a general resistance mechanism employed by *P. aeruginosa* against oxidizing agents.

The EPS matrix is a dynamic and complex space providing the infrastructure for microbial communities like biofilms. Previous research has demonstrated many functions for the EPS matrix, including adhesion, retention of water, nutrient source, exchange of genetic information, and export of cell components, among many others (48). In addition, the EPS also functions as a protective, physical, and chemical barrier, contributing to the increased stress and antibiotic tolerance of biofilms in many bacterial species, including *P. aeruginosa* (5, 15, 21, 44, 48–50), *Staphylococcus* sp (51, 52), and *Escherichia coli* (53, 54). In particular, the polysaccharides and proteins within the EPS matrix are responsible for the increased antimicrobial resistance of biofilms (48).

It has been demonstrated that the polysaccharides Psl and Pel contribute to the increased antimicrobial tolerance of *P. aeruginosa* biofilms. Although their involvement in resistance toward antibiotics, including aminoglycosides, colistin, and ciprofloxacin (15, 21, 44, 49) has been well documented, the effects of Psl and Pel on resistance toward oxidative stress and oxidizing agents such as NaOCl and H_2_O_2_ have been poorly investigated thus far. A recent study found that Psl and Pel are involved in the resistance of *P. aeruginosa* submerged and air-liquid interface biofilms to lethal doses of ultraviolet-A (UVA) radiation, H_2_O_2_, and NaOCl (55). However, this study was predominantly focused on UVA radiation, and results for H_2_O_2_ and NaOCl were limited.

The restricted penetration of molecules into biofilms has been suggested as an important mechanism involved in EPS-mediated resistance (50, 54). For example, tobramycin, a positively charged antibiotic, has been shown to ionically interact with negatively charged components of the biofilm matrix of *P. aeruginosa* at the periphery, impairing its penetration into these structures, an effect that depends on the structure and maturity of the biofilms (56). In accordance with our results, Yang and co-workers showed that treatment with 20 µg/mL tobramycin had the strongest effect on biofilms formed by the Psl- and Pel-deficient PAO1 double mutant ∆*pelA pslBCD*, killing almost all cells within this biofilm after 24 h of treatment. A large portion of cells was also killed in the ∆*pslBCD* biofilms, whereas only some cells in the surface layer of PAO1 WT and ∆*pelA* biofilms were killed by tobramycin (49). Indeed, Billings and colleagues obtained similar results when challenging biofilms of PAO1 WT, a Psl- and a Pel-deficient mutant with antibiotics, with the Psl mutant showing the strongest increase in susceptibility toward tobramycin, colistin, polymyxin B, and ciprofloxacin (21). By adding NaCl to *P. aeruginosa* biofilms, the authors showed that Psl sequesters the positively charged antibiotics colistin, polymyxin B, and tobramycin by electrostatic interactions (21). In addition, it has been previously shown that overproduction of Psl and Pel increased the resistance of *P. aeruginosa* aggregates to tobramycin and ciprofloxacin. In contrast, the deletion of these genes increased the susceptibility of aggregates to these antibiotics. This phenotype was attributed to changes in the bacterial physiology of aggregates, e.g., limited penetration of nutrients and oxygen induced by the matrix components (57). On the other hand, a recent study using an agar-embedded *P. aeruginosa* aggregate model showed that Psl and Pel did not protect *P. aeruginosa* aggregates against tobramycin, ciprofloxacin, and meropenem under these culture conditions (28). The authors suggested that this is most likely due to oxygen limitation and that Psl and Pel affect the metabolic state of the bacteria, which influences antibiotic susceptibility (28). In a different study, Psl production was found to promote resistance of *P. aeruginosa* to the surfactant polysorbate 80 (PS80) (32). Overall, our data are in line with these previous findings about the contribution of the EPS components Psl and Pel to antimicrobial resistance in *P. aeruginosa*.

Interestingly, only the ∆*pslA pelF* double mutant but not the corresponding single Psl and Pel mutants showed a statistically significant difference in susceptibility in the MBC-B assay and cell viability analyses when exposed to sub-lethal concentrations of oxidants in comparison to WT cells. It has been shown that Psl and Pel present redundancy during biofilm development, that is, one polysaccharide can compensate for the lack of the other (25, 33). For example, Ghafoor and colleagues showed that cells of the ∆*pslA* mutant produced significantly more Pel than WT cells (44). It could explain our resistance phenotypes, in which no significant difference was observed when the ∆*pslA* and ∆*pelF* mutants were exposed to sub-lethal concentrations of NaOCl. Further analyses using more defined concentrations of NaOCl showed that Pel increases the resistance of *P. aeruginosa* at 12 µg/mL NaOCl. In accordance with our results (55, 55), we found that the PAO1 ∆*pel,* as well as the double mutant ∆*psl pel*, were consistently more susceptible to NaOCl than the ∆*psl* deficient strain. In our study, the importance of Pel in the NaOCl survival of *P. aeruginosa* was also found for the PA14 strain, indicating that this is not a strain-specific phenotype.

Polysaccharides can be found as cell-associated or cell-free molecules (33). Although it is believed that there is a similarity between these two forms, only the composition of the cell-free form of Pel has been determined (16, 34, 58). The cell-free form of Pel contributes to the mechanical characteristics of *P. aeruginosa* biofilms and reduces *P. aeruginosa* virulence in *Caenorhabditis elegans* and *Drosophila melanogaster* infection models (34). We then showed that the presence of mostly cell-associated Pel (due to the inactivation of PelA hydrolase in the PA14 *pel*^E218A^ strain) did not attenuate the protection against NaOCl, indicating that the form of Pel does not impact NaOCl resistance in *P. aeruginosa*.

We then hypothesized that the exopolysaccharides of the EPS matrix promote resistance to NaOCl by reacting with this oxidant. Indeed, it has been shown that HOCl, the active ingredient of NaOCl, reacts with polysaccharides and sugars, preferably in an N-acetyl group (24, 37, 59, 60). For example, HOCl reacts with hyaluronic acid, and this reaction appears to be localized at the N-acetylglucosamine sugar rings (59). Furthermore, after proteins, the nitrogen-containing amine groups are considered the secondary targets of HOCl (61). These structures and groups can be found in the structure of Pel, which is composed of partially acetylated α−1,4-*N-*acetylgalactosamine comprised predominantly of dimeric repeats of galactosamine and N-acetylgalactosamine (16). The biochemistry of these interactions will be analyzed in more detail in the following study.

The use of glycoside hydrolases encoded in the *psl* and *pel* operon, respectively (PslG_h_ and PelA_h_, respectively), to degrade Psl and Pel has been shown to potentiate the killing effects of antibiotics such as colistin (35), tobramycin, gentamicin, polymyxin B, and neomycin by improving their penetration into the biofilm (36). Furthermore, these enzymes increased neutrophil response against *P. aeruginosa* and the use of topical PslG_h_ to treat wound infections increased bacterial clearance and the antimicrobial effect of tobramycin (36). Additionally, treatment of *P. aeruginosa* and *Staphylococcus aureus* with the glycoside hydrolases cellulase and α-amylase disrupted the biofilms and improved the antimicrobial effect of gentamicin (62). In this study, the treatment with PslG_h_ did not provoke a significant reduction in the susceptibility of PAO1 WT biofilms to NaOCl. Based on our previous results, a slight but not significant reduction in susceptibility upon PslG_h_ treatment followed by NaOCl exposure was expected. Interestingly, degrading Pel with PelA_h_ produced a similar effect, in which no increase in the susceptibility of PAO1 WT biofilms to NaOCl was detected compared with the inactive enzyme control, as observed in our previous analyses using ∆*pel* mutant strains. Since Pel is composed of galactosamine and *N*-acetylgalactosamine (16), we hypothesize that although PelA_h_ cleaves this polysaccharide, amine groups are widely available to quench NaOCl and would still be a substrate for this disinfectant, protecting the biofilms against reactive chlorine stress. Therefore, these results suggest that Pel also protects *P. aeruginosa* biofilms even when the matrix is disrupted.

Growing as multi-species populations is the predominant form of biofilms in nature and confers many advantages for these communities (29). Among them, the species within muti-species populations can share resources such as nutrients and molecules (29) and present increased resistance to antibiotics and disinfectants (63–65). In this context, Psl and Pel play roles in the competition of PAO1 WT and *S. aureus* when grown as dual-species biofilms (55). Furthermore, Psl and Pel produced by *P. aeruginosa* protected *E. coli* and *S. aureus* against the killing effect of colistin (21). In accordance, we found that Psl and Pel produced by PAO1 WT protect non-exopolysaccharides-producing strains such as the ∆*pslA pel* double mutant as well as *E. faecalis*, reinforcing the function of these matrix components as a community good.

The present findings demonstrate that the EPS matrix and the polysaccharide Pel play a role in resistance to oxidizing agents in *P. aeruginosa*. In this context, we hypothesize that the reaction of Pel with NaOCl, for example, could provide biofilm cells more time to develop and activate other defense mechanisms, e.g., synthesis of detoxifying enzymes. Future research will focus on characterizing the exact biochemical interactions of Pel with strong oxidants such as NaOCl and H_2_O_2_ to understand the precise molecular mechanisms of resistance. Understanding the mechanisms by which bacteria escape antimicrobial agents, including strong oxidants, is urgently needed in the fight against antimicrobial resistance and will help in developing new strategies to eliminate resistant strains in any environmental, industrial, and clinical settings.

## MATERIALS AND METHODS

### Bacterial strains and growth conditions

The bacterial strains used in this study are listed in Table S2. Overnight bacterial cultures were grown in LB at 37°C, unless otherwise stated, washed, and diluted in BM2 minimal media [7 mM (NH_4_)_2_SO_4_, 40 mM K_2_HPO_4_, 22 mM KH_2_PO_4_, 0.4% (wt/vol) glucose, 0.5 mM MgSO_4_, 0.01 mM FeSO_4_, pH 7.0]. Biofilms were grown in BM2 biofilm medium [i.e., BM2 supplemented with 0.5% (wt/vol) casamino acids (CAA)] (66, 67) for 24 h at 37°C under static conditions unless otherwise stated. Arabinose at 0.5% (wt/vol) was used to induce the production of Pel in the PAO1 Δ*wspF* Δ*psl* P_BAD_*pel* strain. PA14 cultures and biofilms were grown at 25°C, since lower temperatures support Pel production in PA14. Pel production was monitored in PA14 WT at 25°C and in PAO1 WT at 37°C by dot blot (Fig. S5).

Free chlorine concentration of NaOCl solutions was determined weekly using DPD Free Chlorine Powder Packs (Thermo Scientific Orion) according to the manufacturer’s instructions. Oxidative stress experiments were conducted using BM2 minimal medium to mitigate side reactions between NaOCl and media components. Furthermore, the amount of total RCS remaining in the media after mixing NaOCl and BM2 was determined as previously described (68), and no significant reduction was observed, whereas LB, used as a control, completely quenched NaOCl.

### Minimal bactericidal concentration of biofilms (MBC-B) assay

The MBC-B was determined as described previously (26). Briefly, cells were grown overnight at 37°C at 220 rpm, collected by centrifugation, washed twice, and resuspended in BM2. The bacterial suspension was then diluted in BM2 biofilm medium to an optical density at 600 nm (OD_600nm_) of 0.1 (18) to obtain approximately 1 × 10^8^ CFU/mL. An aliquot of 100 µL was placed into 96-well microtiter plates, and the plates were incubated for 24 h at 37°C under static conditions to allow biofilm formation. The supernatant was removed, and 200 µL of NaOCl (2–1,024 µg/mL) or H_2_O_2_ (1.95–100 mg/mL) diluted in BM2 was added to the pre-formed biofilms. The plates were incubated for 24 h at 37°C, unless otherwise stated, the supernatant was removed, and 100 µL of LB media was added to the wells, followed by incubation for 24 h at 37°C. Finally, the 96-well microplates were stamped and transferred on a 2% (wt/vol) LB agar plate and incubated for 24 h at 37°C. The MBC-B was considered the lowest concentration of oxidizing agent where no bacterial growth was observed.

### Fluorescence microscopy

To visualize the effects of NaOCl and H_2_O_2_ on cells, we conducted fluorescence microscopy using the LIVE/DEAD^TM^ BacLight^TM^ Bacterial viability kit (1:1 Syto9:PI) (Invitrogen^TM^ Thermo Fisher Scientific). Overnight cultures were washed twice and diluted in BM2 biofilm medium to an OD_600nm_ of 0.1 and transferred to flat-bottom 96-well polystyrene microplates, which were incubated under static conditions for 24 h at 37°C to allow adherence and biofilm formation. Biofilms were carefully washed with BM2 minimal medium to remove planktonic cells and treated with NaOCl (4 and 8 µg/mL) or H_2_O_2_ (6.25 and 50 mg/mL) for 1 h at 37°C. Untreated biofilms were used as the growth control. Then, 10 mM of sodium thiosulfate (Na_2_S_2_O_3_) (69) was added to the NaOCl-treated biofilms to quench the oxidizing effect of NaOCl, and the biofilms were subsequently stained with a 1:1 LIVE/DEAD^TM^ BacLight^TM^ and incubated for 15 min in the dark at room temperature (70). Pictures were taken from three independent experiments with three replicates using an EVOS FL Auto 2 microscope (Thermofisher). RFU was quantified using the software Fiji.

### Time-kill kinetics experiments and CFU determination

The viability of *P. aeruginosa* biofilm cells after the treatment with NaOCl was assessed by CFU determination. *P. aeruginosa* strains were grown overnight in LB at 37°C and 220 rpm, washed twice, and resuspended in BM2 biofilm medium to an OD_600nm_ of 0.1. Aliquots of 1 mL were transferred to 12-well microtiter plates and incubated for 24 h at 37°C under static conditions to allow biofilm formation. *P. aeruginosa* PA14 strains were grown at 25°C. Then, the media was removed, and biofilms were carefully washed with BM2 and treated with NaOCl for 1 h at 37°C. Na_2_O_2_S_3_ at 10 mM was added to the samples to quench the effect of the remaining NaOCl (69), and biofilm cells were collected by scraping. Untreated biofilms were used as the positive control. Samples were serially diluted and plated out on LB agar plates using the drop plate method (71).

### Co-culture biofilms of *P. aeruginosa* PAO1 WT with *E. faecalis* and ∆*pslA pelF*

*Co-culture of PAO1 WT (pJN105) and ∆pslA pelF (pUCP20*): Plasmids pJN105 and pUCP20 were transferred into PAO1 WT and ∆*pslA pelF*, respectively, by electroporation (72). *P. aeruginosa* was grown overnight in LB containing 30 µg/mL gentamicin or 300 µg/mL carbenicillin, respectively. Cells were collected by centrifugation, washed twice, and resuspended in BM2. Bacterial suspensions were diluted in BM2 biofilm medium to an OD_600nm_ of 0.05. Mixed biofilms were produced by mixing 1:1 of 0.05 OD_600nm_ of PAO1-pJN105 and ∆*pslA pelF*-pUCP20. Monoculture biofilms were prepared and used as controls. One milliliter of mono or mixed cultures was transferred to 12-well microplates, which were incubated for 24 h at 37°C under static conditions to allow biofilm establishment and treated with NaOCl at 8 µg/mL or H_2_O_2_ at 6.25 mg/mL for 1 h at 37°C. Na_2_S_2_O_3_ was added to the NaOCl-treated biofilms, the cells were scraped, and the CFU/well was determined by the drop plate method (71). PAO1 WT was selected by growth on agar plates containing 30 µg/mL gentamicin, and ∆*pslA pelF* was selected by growth on agar plates with 300 µg/mL carbenicillin.

*Co-culture of PAO1 WT or ∆pslA pelF with E. faecalis*: Overnight cells were collected by centrifugation and washed twice with PBS. *E. faecalis* was diluted in DMEM to an OD_600nm_ of 0.01. PAO1 WT and *∆pslA pelF* were diluted to an OD_600nm_ of 0.05, followed by a 1/1,000 dilution. Mixed biofilms were prepared by mixing *E. faecalis* at 0.01 OD_600nm_ with a 1/50,000 dilution of PAO1 WT or *∆pslA pelF* in DMEM. Monocultures were prepared and used as controls. One milliliter of mixed cultures or monocultures was added to 12-well microplates, which were incubated for 24 h at 37°C and static conditions. The biofilms were then washed and treated with NaOCl at 8 µg/mL and H_2_O_2_ at 6.25 mg/mL for 1 h at 37°C. Na_2_S_2_O_3_ was added to the NaOCl-treated biofilms, which were scraped, and the CFU/well was determined by the drop plate method (71). LB agar plates supplemented with 5 µg/mL gentamicin and 12 µg/mL polymyxin B were used to select *E. faecalis,* and *P. aeruginosa* was selected using BM2 agar plates.

### Colony biofilm assay

Colony biofilm assay was performed as previously described (27, 73), with slight modifications. *P. aeruginosa* PAO1 WT, ∆*pslA,* ∆*pelF,* and ∆*pslA pelF* were grown overnight in LB media under shaking conditions (220 rpm) at 37°C. Then, overnight bacterial cells were harvested by centrifugation and washed, and the OD_600nm_ was adjusted to 0.05 (5 × 10^7^ CFU/mL) in PBS. Then, 25 mm polycarbonate membranes with a pore size of 0.22 µm were placed in agar plates using sterilized forceps. Ten microliters of bacterial culture were inoculated in the center of the semi-permeable membranes, and the plates were incubated for 48 h at 37°C. Then, using sterile forceps, the membranes were removed from the agar plates and transferred to 50 mL Falcon tubes containing PBS for the untreated control or NaOCl at 32 µg/mL, which were incubated for 1 h at 37°C. Ten mM Na_2_S_2_O_3_ was added to quench the toxic effect of NaOCl, and cells were vigorously vortexed for two pulses of 1 min each. Serial dilutions were prepared, and the samples were plated out on LB agar plates following the drop plate method (71).

### Recombinant GH expression and purification

His-tagged recombinant PelA_h_ and PslG_h_ were expressed in Clearcoli^®^ cells grown in Terrific Broth (Bioshop) or autoinduction medium with 50 µg/mL Kanamycin (Biobasic) as previously described (35, 74). Bacterial cultures in Terrific Broth were induced with 0.5 mM isopropyl-*β*-D-thiogalactopyranoside (IPTG) (Biobasic) when the cells reached an optical density at 600 nm (OD_600_) of 1.2 to 1.4. The cells were incubated post-induction overnight at 18°C with shaking at 200 rpm before being harvested by centrifugation at 5,000 × *g* for 30 min at 4°C. Both proteins were purified using Ni-nitrilotriacetic acid columns (GE Healthcare) followed by buffer exchange as previously described (74).

### Treatment of biofilms with hydrolases

We investigated the effect of NaOCl on *P. aeruginosa* PAO1 biofilms after degrading Psl and Pel with the glycoside hydrolases PslG and PelA (PslG_h_ and PelA_h_, respectively) (36). PAO1 overnight cells were washed, the OD_600nm_ was adjusted to 0.1 (1 × 10^8^ CFU/mL) in BM2 biofilm medium, and 1 mL was transferred to 12-well microtiter plates for 24 h at 37°C under static conditions. Then, biofilms were carefully washed and treated with 2 µM PslG_h_ or PelA_h_ for 1 h at 25°C, followed by treatment with NaOCl at 12 µg/mL for 1 h at 37°C. Na_2_O_2_S_3_ at 10 mM was added to the samples to quench the effect of the remaining NaOCl (69), and biofilm cells were collected by scraping and plated out on LB agar plates following the drop plate method (71). Heat-inactive enzymes were used as controls.

### Statistical analyses

All experiments were performed in at least three independent experiments. Data are presented as log_10_ reduction (75, 76) calculated based on the untreated and NaOCl-treated conditions and compared with PAO1 or PA14 WT data, which were used as the positive control. Statistical analyses were performed using GraphPad Prism software version 9.0 (San Diego, USA). The data are expressed as mean ± standard deviation (SD). Data normality was confirmed by the Shapiro-Wilk or D'Agostino-Pearson test. Then, parametric data were analyzed by one-way ANOVA, followed by Tukey or Dunnett’s post-test for multiple comparisons or Student’s *t*-test for comparison between two groups. On the other hand, non-parametric distribution was evaluated by the *t*-test and Mann-Whitney test for comparison between the two groups. Results were considered statistically significant when *P* < 0.05.

## Data Availability

The authors confirm that the data supporting the findings of this study are available within the article.

## SUPPLEMENTAL MATERIAL

Supplemental material spectrum.00922-24-s0001.docx Supplemental tables, figures, and methods.

ASM does not own the copyrights to Supplemental Material that may be linked to, or accessed through, an article. The authors have granted ASM a non-exclusive, world-wide license to publish the Supplemental Material files. Please contact the corresponding author directly for reuse.
