# Supplementary material for: The role of exopolysaccharides Psl and Pel in resistance of Pseudomonas aeruginosa to the oxidative stressors sodium hypochlorite and hydrogen peroxide
Source: Microbiol Spectr. 2024 Aug 28;12(10):e00922-24. doi: 10.1128/spectrum.00922-24 (PMC11448232; doi:10.1128/spectrum.00922-24)
Supplement: Supplemental material — Supplemental tables, figures, and methods. [file spectrum.00922-24-s0001.docx]

**SUPPLEMENTAL MATERIAL**

**Tables**

**Supplementary Table 1:** Area under the curve (AUC) of growth curves of *P. aeruginosa* PAO1 WT and Δ*pslA,* Δ*pelF* and Δ*pslA pelF* at different concentrations of NaOCl.

|  | NaOCl concentrations (µg/mL) | | | |
| --- | --- | --- | --- | --- |
|  | **Untreated** | **0.5** | **1** | **2** |
| Δ*pslA* | 7.872 ± .450 | 7.465 ± .640 | 5.092 ± 2.115 | 1.9 ± 0.072 |
| Δ*pelF* | 8.260 ± 0.212 | 8.126 ± 0.244 | 6.680 ± 0.824 | 1.979 ± 0.054 |
| Δ*pslA pelF* | 8.471 ± 0.380 | 8.130 ± 0.513 | 4.430 ± 0.871 | 1.937 ± 0.050 |
| PAO1 WT | 8.001 ± 0.841 | 7.904 ± 0.644 | 6.307 ± 1.146 | 1.981 ± 0.052 |

**Supplementary Table 2:** List of strains used in this study.

| Strain | Relevant characteristics | Source |
| --- | --- | --- |
| *Pseudomonas aeruginosa* PAO1 strains | | |
| PAO1 WT | Wild-type strain | (1) |
| PAO1 ∆*pslA* | Psl-deficient mutant of PAO1 WT | (2) |
| PAO1 ∆*pelF* | Pel-deficient mutant of PAO1 WT | (2) |
| PAO1 ∆*pslA pelF* | Psl- and Pel-deficient mutant of PAO1 WT | (2) |
| PAO1 (pJN105) | PAO1 WT harboring the empty vector pJN105; Gen resistance | This study |
| ∆*pslA pelF* (pUCP20) | Psl- and Pel-deficient mutant of PAO1 WT harboring the empty vector pUCP20; Carb resistance | This study |
| PAO1 Δ*wspF* Δ*psl* P_BAD_*pel* | In-frame deletion of *wspF* and *psl*, araC-P_BAD_ inserted upstream of *pel* | (3) |
| *Pseudomonas aeruginosa* PA14 strains | | |
| PA14 WT | Wild-type strain | (3) |
| PA14 ∆*pslA* | Pel-deficient mutant of PA14 WT | (4) |
| PA14 ∆*pelF* | Pel-deficient mutant of PA14 WT | (4) |
| PA14 *pelA*^E218A^ | PA14 mutant strains with abrogated PelA hydrolase activity produced using the inactive catalytic point variant E218A | (4) |

Gen: gentamycin; Carb: carbenicillin

**Figures**

**A**

**
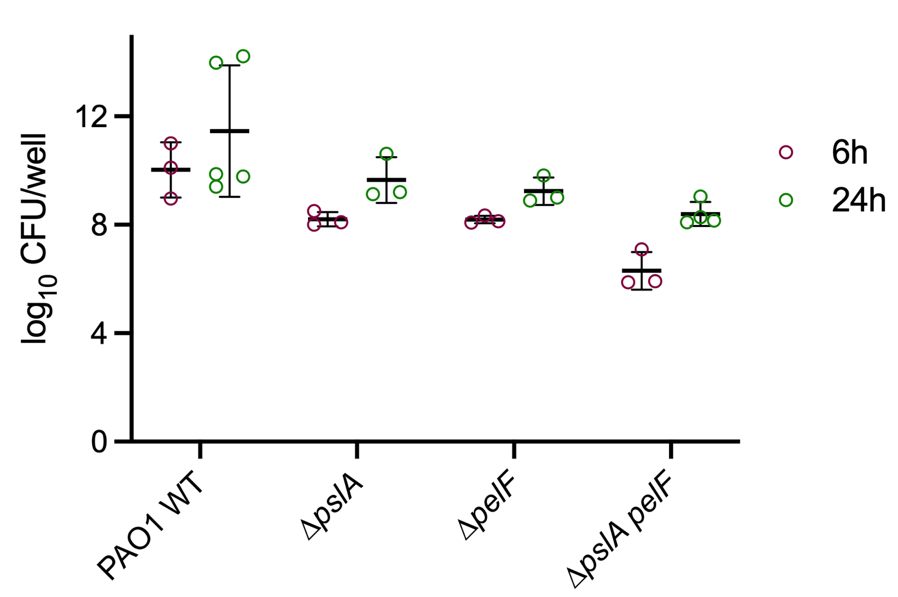
**

**B**

**
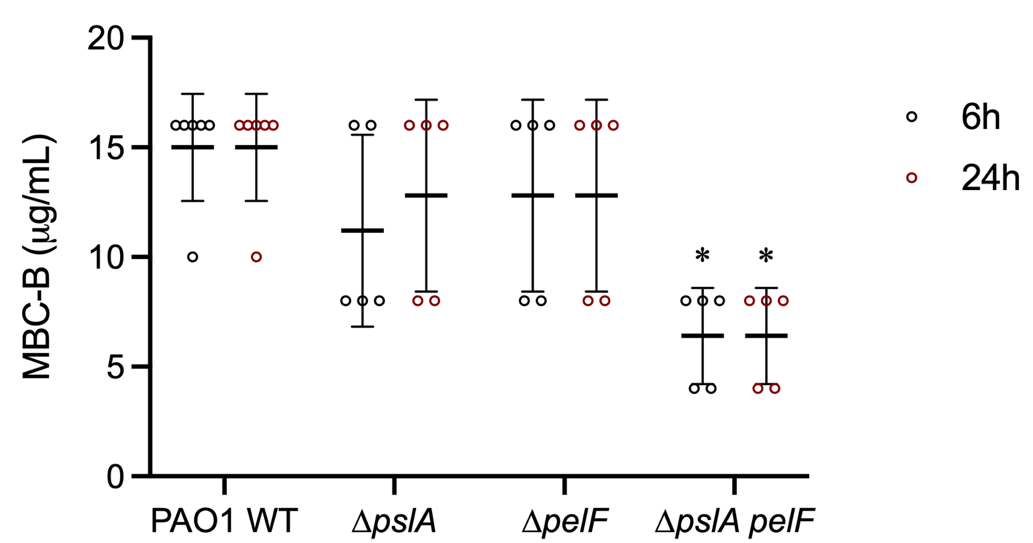
**

**Supplementary Figure 1:** Susceptibility of PAO1 WT and PAO1 ∆*pslA,* ∆*pelF,* and ∆*pslA pelF* biofilms grown for 6 and 24 h. (A) CFU analyses of biofilms grown for 6 and 24 h. PAO1 WT and mutants were grown in BM2 supplemented with 0.5% CAA for 6 and 24h at 37^o^C under static conditions, and CFU/mL was determined by the drop-plate method. (B) Effect of NaOCl on *P. aeruginosa* PAO1 WT and mutant strains by the MBC-B assay. PAO1 WT and mutants were grown in BM2 biofilm medium for 6 and 24 h at 37^o^C under static conditions and treated with NaOCl (2 – 1,024 μg/mL) for 1 h. Results represent the mean and standard deviation of at least three independent experiments (* p < 0.05).

| **A** | **B** |
| --- | --- |
| 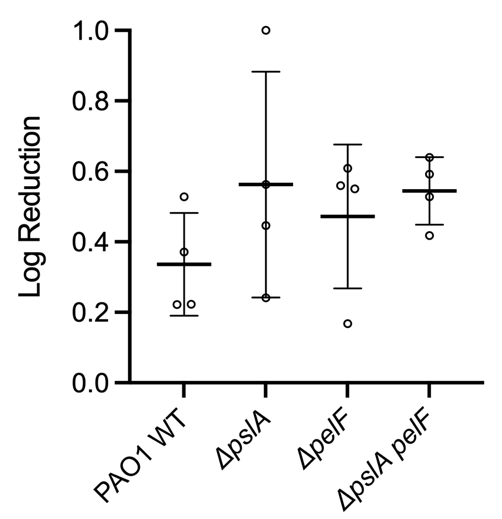 | 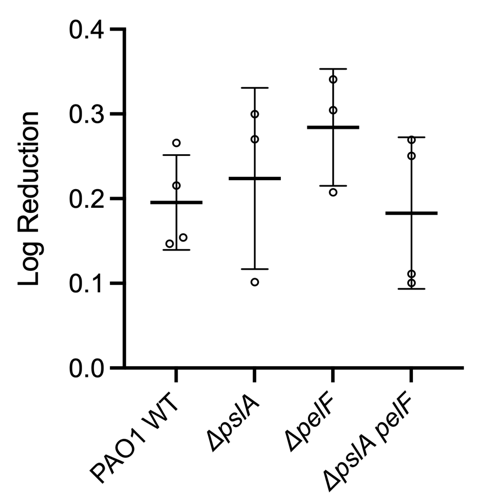 |

**Supplementary Figure 2:** Susceptibility of PAO1 ∆*pslA,* ∆*pelF,* ∆*pslA pelF,* and PAO1 WT planktonic cells to NaOCl. Overnight cultures were grown in BM2 at 37^o^C and 220 rpm. Then, two approaches were used: (A) cells were washed twice, and resuspended in BM2 minimal medium, and (B) overnight cells were diluted in BM2 minimal media. Planktonic cells were treated with NaOCl at 1.25 μg/mL (final cell concentration of 1 × 10^8^ CFU/mL) for 1 h at 37^o^C. Then, 10 mM sodium thiosulfate was added to quench the toxic effect of the remaining NaOCl, and CFU was determined by the drop method. Data represent the mean and standard deviation of at least three independent experiments.

**A**


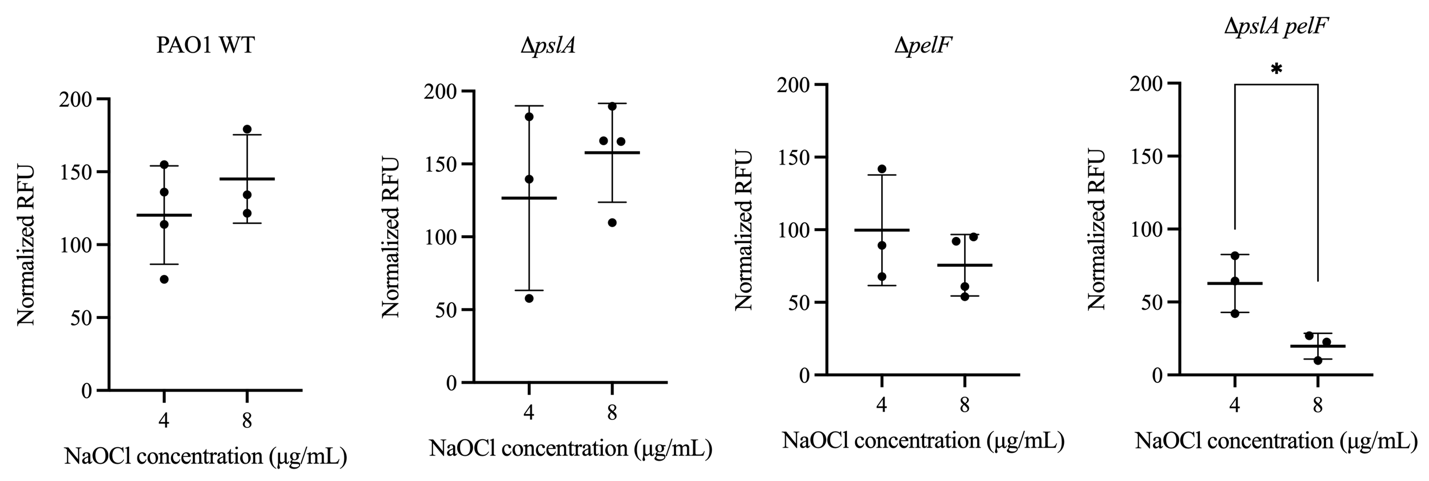


**B**

**
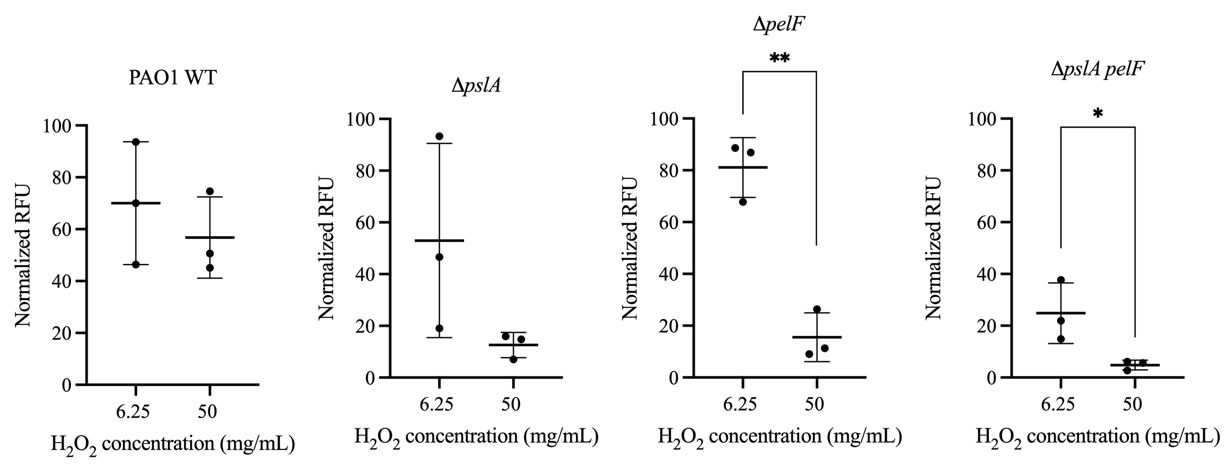
**

**Supplementary Figure 3:** Quantification of live cells in (A) NaOCl or (B) H_2_O_2_-treated biofilms. Biofilms were grown in flat-bottom polystyrene 96-well microplates, treated with NaOCl at 4 or 8 μg/mL NaOCl or H_2_O_2_ at 6.25 and 50 mg/mL for 1 h at 37^o^C under static conditions, and stained with DNA-intercalating 1:1 Syto9 and PI dyes and visualized by fluorescence microscopy. Relative fluorescence units (RFU) were determined by Fiji, and the data were normalized based on the untreated control. Results represent the mean and standard deviation of at least three independent experiments. (* p < 0.05).


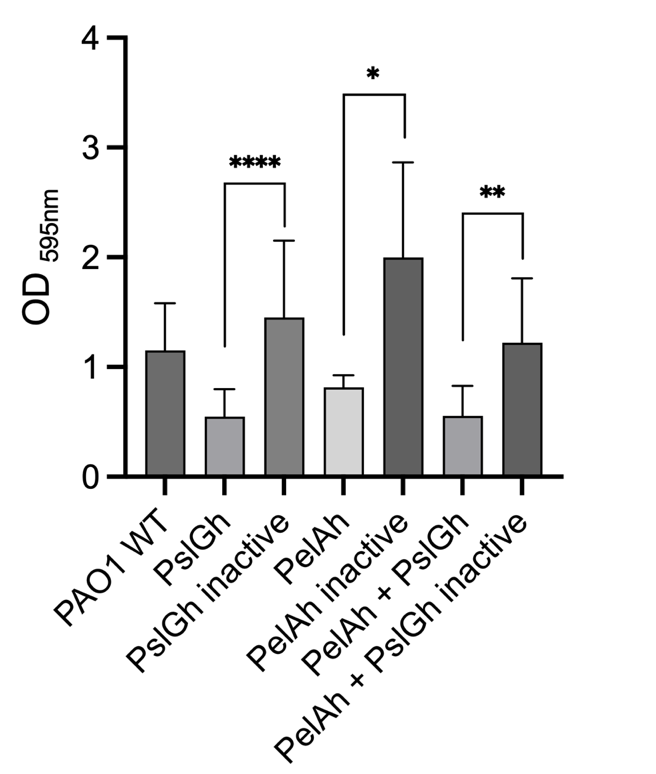


**Supplementary Figure 4:** Biofilm biomass quantification of PAO1 WT biofilms treated with PslG_h_, PelA_h_, or PslG_h_ and PelA_h_. PAO1 WT biofilms without hydrolase treatment were also prepared. PAO1 WT biofilms were grown in 96-well microplates on BM2 biofilm medium for 24 h at 37^o^C under static conditions. Then, the biofilms were washed, and 2 μM of PslG_h_, PelA_h_, or the combination of PslG_h_ and PelA_h_ for 1 h. The media was removed, and biofilms were washed and stained with 0.1% (wt/vol) crystal violet solution for 10 min. Biofilm quantification was performed by OD reading at 595 nm using an Epoch plate reader. Results represent the mean and standard deviation of at least three independent experiments. (* p < 0.05; ** p < 0.01; **** p < 0.0001).


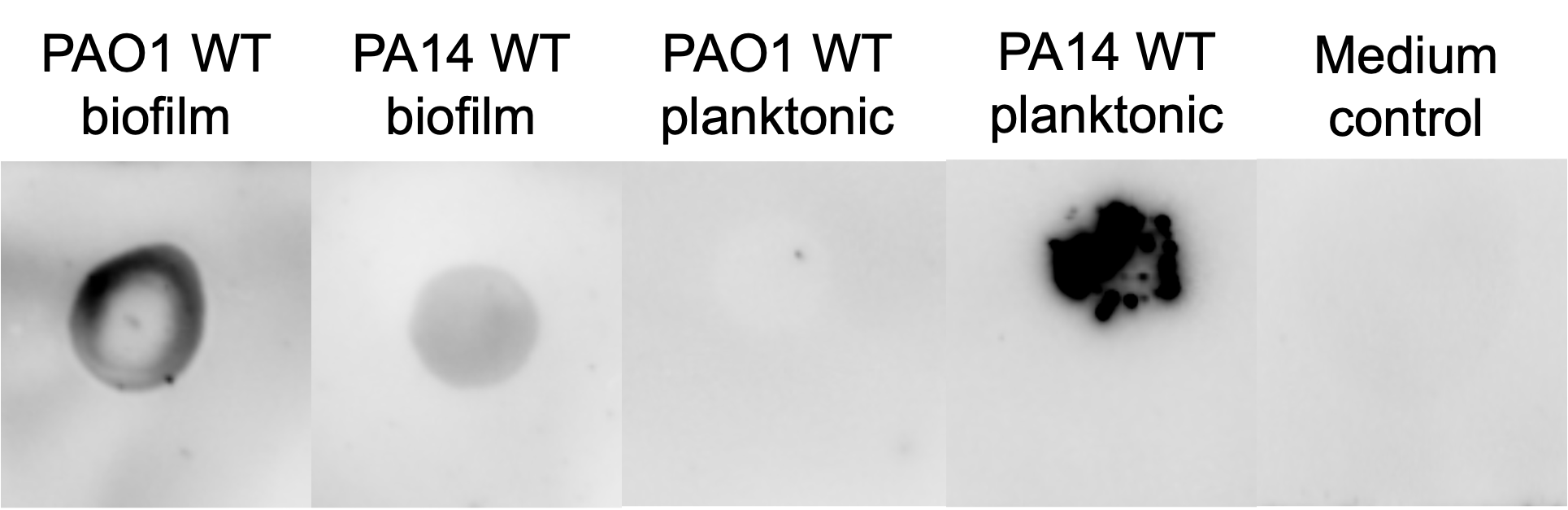


**Supplementary Figure 5:** Pel production by PAO1 WT and PA14 WT biofilms. Biofilms were grown in petri dishes for 24 h in BM2 biofilm medium at 37^o^C for PAO1 WT and 26.5^o^C for PA14 WT. Planktonic cells were grown overnight in BM2 biofilm medium at 37^o^C for PAO1 WT and 25^o^C for PA14 WT under shaking conditions. Cells were scraped and collected by centrifugation at 16,000 x g and 2 min, the pellet was resuspended in EDTA and boiled for 20 min. Then, the supernatant was collected by centrifugation at 16,000 x g for 10 min and treated with Proteinase K for 1 h at 60^o^C, followed by incubation at 80^o^C for 30 min. Pel detection was conducted using the lectin *Wisteria floribunda* (WFL) conjugated with horseradish peroxidase (HRP). PAO1 WT planktonic cultures grown for 24 h at 37^o^C in BM2 biofilm medium (no production of pel under planktonic growth in PAO1) and BM2 medium control were used as the controls.

**Materials and Methods**

**Growth kinetics**

PAO1 WT, ∆*pslA,* ∆*pelF,* and ∆*pslA pelF* grown overnight in LB at 37^o^C under shaking conditions were collected by centrifugation, washed twice, and resuspended in BM2. Then, NaOCl at 0.5, 1, 2 was added to bacterial cells to a final concentration of 1 x 10^8^ CFU/mL. The OD_600nm_ was recorded every hour for 20 h at 37^o^C by an Epoch plate reader.

**CFU determination**

The viability of P. aeruginosa planktonic cells after the treatment with NaOCl was assessed by CFU determination. *P. aeruginosa* was grown overnight in BM2 at 37^o^C under shaking conditions. Then, two approaches were used: (i) overnight cells were collected by centrifugation, washed twice, and resuspended in BM2, and (ii) 1 mL of overnight cultures were transferred to microcentrifuge tubes, and no washing step was performed. The OD_600nm_ was adjusted to 0.1 (1 x 10^8^ CFU/mL), and the bacterial suspensions were treated with NaOCl at 1.25 μg/mL. Then, 10 mM Na_2_S_2_O_3_ (5) was added to the planktonic-treated samples to quench the NaOCl effect, serial dilutions were prepared, and 5 x 10 μL of the dilutions were plated out on 1.5% LB agar plates following the drop plate method (6). The plates were incubated for 24 h at 37oC, cells were counted, and the CFU/well was determined.

**Crystal violet staining**

PAO1 WT biofilms were grown in 96-well microplates on BM2 biofilm medium for 24 h at 37^o^C under static conditions, washed and treated with 2 μM of PslG_h_, PelA_h_, or the combination of PslG_h_ and PelA_h_ for 1 h. PAO1 WT biofilms without hydrolase treatment were also prepared. The media was removed, and biofilms were washed and stained with 0.1% (wt/vol) crystal violet solution for 10 min. The wells were washed with H_2_O to remove excess crystal violet, and 95% (vol/vol) ethanol was added for 20 min. Biofilm quantification was performed by OD reading at 595 nm using an Epoch plate reader.

**WFL-HRP Dot Blots**

Pel detection in biofilms was performed as previously described (4). Briefly, *P. aeruginosa* PAO1 and PA14 WT biofilms were grown in petri dishes in BM2 biofilm medium at 37^o^C and 26.5^o^C, respectively, for 24 h and static conditions. Planktonic cells were grown overnight in BM2 biofilm medium at 37^o^C for PAO1 WT and 25^o^C for PA14 WT under shaking conditions. Then, to detect Pel, 1 mL of cells scraped from the plates were collected by centrifugation, resuspended in EDTA, and boiled for 20 min. Cultures were centrifuged for 2 min at 16,000 x g, and the supernatant was collected and treated with Proteinase K, followed by a 1-h incubation at 60^o^C and 30 min at 80^o^C. For this, the pellet of 1 mL of 24 h cultures were collected by centrifugation for 2 min at 16,000 x g, resuspended in EDTA, boiled, and treated with Proteinase K as described above.

After sample preparation, 7.5 µL of these samples were pipetted onto a nitrocellulose membrane and left to air dry for 15 min. The membrane was then blocked with 5% (w/v) bovine serum albumin (BSA) in Tris-buffered saline with 0.5% (v/v) Tween-20 (TBST) for 2 h shaking at room temperature, followed by an overnight shaking incubation at 4^o^C with 10 μg/mL of the lectin Wisteria floribunda (WFL) conjugated with horseradish peroxidase (HRP) (WFL-HRP; EY Laboratories) in 2% BSA in TBST with 0.2 g/L calcium chloride. The membrane was washed twice with TBST for 10 min and subsequently imaged using Supersignal West Pico Substrate (Thermo Scientific).

**References:**

1. Stover CK, Pham XQ, Erwin AL, Mizoguchi SD, Warrener P, Hickey MJ, Brinkman FSL, Hufnagle WO, Kowalik DJ, Lagrou M, Garber RL, Goltry L, Tolentino E, Westbrock-Wadman S, Yuan Y, Brody LL, Coulter SN, Folger KR, Kas A, Larbig K, Lim R, Smith K, Spencer D, Wong GK-S, Wu Z, Paulsen IT, Reizer J, Saier MH, Hancock REW, Lory S, Olson MV. 2000. Complete genome sequence of Pseudomonas aeruginosa PAO1, an opportunistic pathogen. 6799. Nature 406:959–964.

2. Ghafoor A, Hay ID, Rehm BHA. 2011. Role of Exopolysaccharides in Pseudomonas aeruginosa Biofilm Formation and Architecture. Appl Environ Microbiol 77:5238–5246.

3. Colvin KM, Alnabelseya N, Baker P, Whitney JC, Howell PL, Parsek MR. 2013. PelA Deacetylase Activity Is Required for Pel Polysaccharide Synthesis in Pseudomonas aeruginosa. J Bacteriol 195:2329–2339.

4. Razvi E, Whitfield GB, Reichhardt C, Dreifus JE, Willis AR, Gluscencova OB, Gloag ES, Awad TS, Rich JD, da Silva DP, Bond W, Le Mauff F, Sheppard DC, Hatton BD, Stoodley P, Reinke AW, Boulianne GL, Wozniak DJ, Harrison JJ, Parsek MR, Howell PL. 2023. Glycoside hydrolase processing of the Pel polysaccharide alters biofilm biomechanics and Pseudomonas aeruginosa virulence. 1. Npj Biofilms Microbiomes 9:1–14.

5. Groitl B, Dahl J-U, Schroeder JW, Jakob U. 2017. Pseudomonas aeruginosa defense systems against microbicidal oxidants. Mol Microbiol 106:335–350.

6. Herigstad B, Hamilton M, Heersink J. 2001. How to optimize the drop plate method for enumerating bacteria. J Microbiol Methods 44:121–129.
